# The Associations Between Oxytocin and Trauma in Humans: A Systematic Review

**DOI:** 10.3389/fphar.2018.00154

**Published:** 2018-03-01

**Authors:** Mariana Fortunata Donadon, Rocio Martin-Santos, Flávia de Lima Osório

**Affiliations:** ^1^Department of Neuroscience and Behavior, Medical School of Ribeirão Preto, University of São Paulo, São Paulo, Brazil; ^2^Department of Psychiatry and Psychology, Hospital Clínic, Institut d'Investigacions Biomèdiques August Pi i Sunyer (IDIBAPS), Centro de Investigación en Red de Salud Mental (CIBERSAM), Barcelona, Spain; ^3^Department of Medicine, University of Barcelona, Barcelona, Spain; ^4^Technology Institute for Translational Medicine (INCT), National Council for Scientific and Technological Development (CNPq), Brasília, Brazil

**Keywords:** oxytocin, early trauma, current trauma, PTSD, systematic review, PRISMA, qualitative assessment

## Abstract

Studies have shown that traumatic experiences may affect hormonal systems mediated by the hypothalamic-pituitary-adrenal (HPA) axis and the oxytocinergic system. This effect is the result of long-term impairments in hypothalamic structures and negative feedback mechanisms within the HPA axis, structures that mediate the response to stress. This deregulation reduces the production and release of cortisol and oxytocin (OXT), which may alter stress responses and lead to increased vulnerability to impairments from stressful experiences. The presence of gene polymorphisms might also have an impact on the vulnerability to psychopathology. We made a systematic review of articles dealing with the relationship between OXT and traumatic emotional experiences in humans. Thirty-five studies were reviewed and significant associations between experiences of emotional trauma (ET) and OXT were found. The main results showed that the presence of ET and post-traumatic stress disorder (PTSD) is strongly associated with reductions in endogenous OXT, and also that the acute effects of OXT administrations in individuals with ET tend to be anxiolytic only in less severe forms. In victims of recent traumatic experiences (RTE), OXT increased the re-experience of traumas and restored the function of different neural networks associated with fear control/extinction in PTSD patients. The results available also suggest that gene receptor polymorphisms may have a protective function in different outcomes after the experience of traumatic events. We conclude that the relationship between ET and OXT is multifaceted, complex, and mediated by contextual and individual factors. Directions for future studies are suggested considering the gaps in the available literature.

## Introduction

Traumatic and stressful experiences throughout life, whether acute or chronic, may lead to changes in different bodily systems that increase the vulnerability to psychopathology (Meewisse et al., 2007; McQuaid et al., 2016). One of the most well-known of such changes concerns hormonal systems, including the hypothalamic-pituitary-adrenal (HPA) axis. The HPA axis plays a fundamental role in responding to both external and internal stimuli, including psychological stressors, and is also believed to be implicated in vulnerability to mental illnesses (Heinrichs et al., 2003; Juruena et al., 2004; Boyce and Ellis, 2005; Neumann and Landgraf, 2012; Olff, 2012; Kuhlman et al., 2015).

When facing stressful situations, especially during the early stages of development, the HPA axis can be either hypo- or hyperactived, with the possibility of excessive exposure to glucocorticoids and their deleterious effects. These effects can persist throughout the lifespan because as the HPA axis may remain unstable, hypersensitive, or dysfunctional. This contributes to the weakening of the immune system, to increased vulnerability to different physical and mental illnesses, and to the inability to cope with subsequent stressful/traumatic events that may lead to exhaustion of the organism (Mirescu et al., 2004; Smith and Vale, 2006; Faravelli et al., 2012).

Several neurotransmitters and neuropeptides also affect the function of the HPA axis, including oxytocin (OXT). OXT is a neurohormone produced in the hypothalamus by the supraoptic and paraventricular nuclei. It is sent to the posterior pituitary or neurohypophysis and, from there, it is secreted into the bloodstream to produce its effects (Gimpl and Fahrenholz, 2001). OXT has peripheral and central functions and its action in breastfeeding, childbirth and maternal behavior is well established (Ring et al., 2006; Yoshida et al., 2009; Neumann and Landgraf, 2012).

In addition to these functions, OXT has therapeutic potential associated with the promotion of pro-social behaviors such as increased self-confidence, positive social memories, and affiliative behavior. Furthermore, previous studies have investigated the possibility that the administration of OXT may lead to reductions in anxiety and stress levels (Savaskan et al., 2008; Guastella et al., 2009; Rimmele et al., 2009; Ross and Young, 2009; Fischer-Shofty et al., 2010).

Existing evidence suggests that central OXT release contributes to the modulation and maintenance of cortisol levels that favor the rapid return of the body to its pre-stress baseline state (Amico et al., 2004; Gulpinar and Yegen, 2004; Heinrichs et al., 2004) to minimize the response of the HPA axis to psychologically stressful stimuli. However, stressful experiences might also alter the functioning of the suprachiasmatic nucleus, decreasing the synthesis and release of endogenous OXT (Ozbay et al., 2008; Gonzalez et al., 2009; Nicolson et al., 2010). As result, the negative feedback mechanism of the HPA axis may be affected, leading to hypercortisolemia (Yehuda et al., 2000; Brown et al., 2016). On the behavioral level, the decrease in endogenous OXT levels reduces the multiple pro-social functions of this hormone, thereby reducing coping and resiliency responses (Opacka-Juffry and Mohiyeddini, 2012; Frijling et al., 2015).

In light of the above findings, the release of exogenous administration of OXT can reduce hormonal and subjective responses to stress, reduce cortisol release in the blood stream, and reestablish bodily homeostasis, therefore placing OXT as a potential therapeutic agent (Cardoso et al., 2013, 2014). Another line of research deals with the role that genetic polymorphisms play in OXT receptor genes, which might alter the individual vulnerability to stress since changes in receptor configuration can reduce or prevent the binding of OXT and its consequent effects in the body (Champagne and Curley, 2009; Skuse and Gallagher, 2011; Unternaehrer et al., 2012; Bakermans-Kranenburg and van IJzendoorn, 2014).

As seen, a number of recent studies have attempted to provide a detailed understanding of the multiple and complex associations between OXT and different traumatic and stressful situations, whether chronic or acute (Myers et al., 2014; Seltzer et al., 2014; Mizushima et al., 2015; Van Zuiden et al., 2017). The objective of the current study was to systematically review this literature to highlight the major contributions of the studies conducted to date dealing with the associations between OXT and traumatic emotional experiences in humans.

## Methodology

The guidelines of the Preferred Reporting Items for Systematic Reviews and Meta-Analyses (PRISMA—Moher et al., 2009) were adopted as the methodological framework of this study. The electronic databases PsycINFO, PubMed, Scielo, Web of Science, and LILACS were searched without limitations in terms of time, language, or publication date (last search conducted on December 3, 2017). A manual search of the reference lists of the selected articles was also performed. The following keywords were used followed by “AND” or “OR:” oxytocin; PTSD; post-traumatic stress; early trauma; childhood maltreatment; emotional trauma; emotional stress; neglect; adversity; sexual abuse; emotional abuse; and physical abuse.

Articles that evaluated associations between OXT and the experience of early trauma (ET), recent traumatic experiences (RTE), and post-traumatic stress disorder (PTSD) in men and women, regardless of age, were eligible for inclusion. ET experiences were considered as those that occurred before 18 years of age (Bremner et al., 2000), and RTEs were considered as those that occurred over the last few days or months (Chatzittofis et al., 2014).

Studies of endogenous OXT that did not involve participants with ET, RTE, or PTSD; those investigating OXT and psychiatric disorders excluding PTSD; those involving OXT and social behavior, resilience, social cognition, genetic analysis, and the physiological aspects of the HPA axis among participants without ET, RTE, or PTSD; those involving treatment for ET or PTSD but not involving OXT; and animal studies, letters to the editor, case studies, and reviews unrelated to this topic were excluded.

Two researchers made independent decisions regarding whether to include a study, and divergences in data extraction were discussed until a consensus was reached.

**Figure 1:**
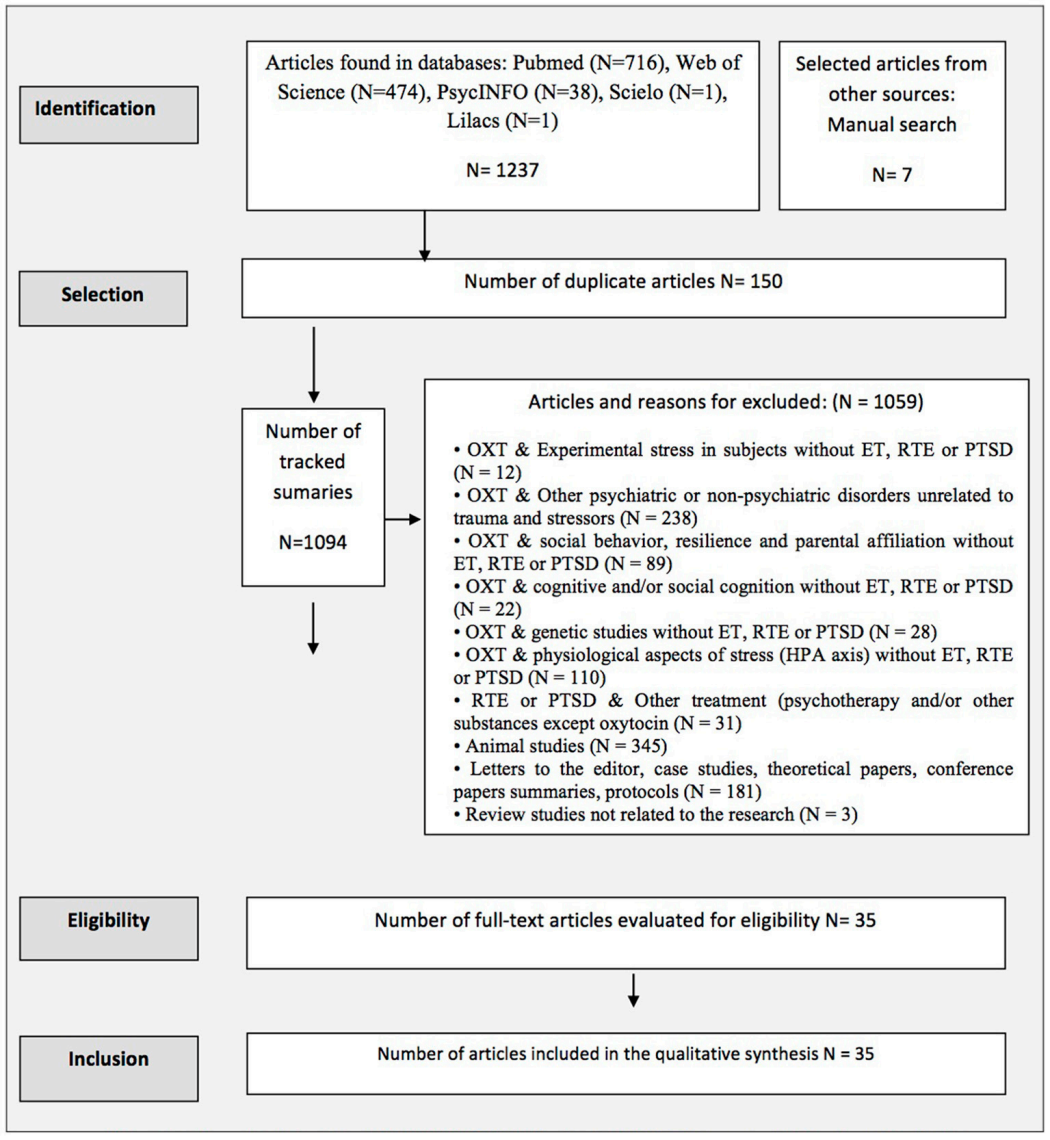
Flowchart based on PRISMA/(OXT, Oxytocin; ET, Early trauma; HPA, Hypothalamic Legend Pituitary Adrenal Axis; PTSD, Post traumatic stress disorder; RTE, Recent trauma event).

Figure 1 shows the search results as well the reasons for article exclusion.

The methodological quality of the studies was evaluated using the following references: (a) Strengthening the Reporting of Observational Studies (STROBE; Von Elm et al., 2007); (b) Transparent Reporting of Evaluations with Non-Randomized Designs (TREND; Des Jarlais et al., 2004); and (c) the Revised Recommendations for Improving the Quality of Reports of Parallel-Group Randomized Trials (CONSORT; Moher et al., 2001). The quality percentage of each study was calculated by dividing the number of items scored by the total number of items.

## Results

A total of 1,244 articles were found, and 35 articles were selected after applying the inclusion and exclusion criteria (see Figure 1).

Table 1 below presents the main characteristics of the included studies in relation to the samples, outcomes assessment, and methodological quality. For more details, see the Excel dataset in the Supplementary Material (S1).

**Table 1:** Characteristics of the studies included in this review.

**Author/Year**	**Study design**	**Interest sample**				**Comparison sample**				**Outcomes assessment**			Quality assessment^**^
		**N/** **Sex**	**Age Mean/range**	**Type**	**Trauma**	**N/** **Sex**	**Age** **Mean/range**	**Type**	**Trauma**	[OXT]^*^	**Other variables/** **Instrument**	**Trauma tool**	**%**
**1 - OBSERVATIONAL STUDIES—ENDOGENOUS OXYTOCIN**													**Mean: 76.28%**
Heim et al., 2009	Cross-sectional	22**F**	18-45	Outpatients Healthy Institution	ET	—	—	—	—	CSF/EIA kit	—	CTQ	67.0
Opacka-Juffry and Mohiyeddini, 2012	Cross-sectional	90**M**	18-56	Healthy General Population	ET RTE	—	—	—	—	Blood /RIA	—	ELS-C, ELS-A, RSLEs	68.0
Chatzittofis et al., 2014	Cross-sectional	18**M**	23-65	Inpatients Suicide Attempters	ET+ RTE /Non- RTE	—	—	—	—	CSF/RIA	—	KIVS, Interview	74.0
Mohiyeddini et al., 2014	Cross-sectional	90**M**	18-56	Healthy General Population	ET	—	—	—	—	Blood/RIA	—	ELS- C	85.0
Mizuki and Fujiwara, 2015	Cross-sectional	31**M** 49**F**	18-48	Healthy General Population	ET/Non-ET	—	—	—	—	Urine/ASKA kit	—	CTQ	77.0
Frijling et al., 2015	Case-Control	21**M** 19**F**	22-59	Outpatients Police Personal Clinic	PTSD + ET/Non-ET	20**M** 20**F**	22–59	Police officers	RTE + ET/Non-ET	Saliva/RIA	—	CAPS, SCID ETI	90.0
Mizushima et al., 2015	Case-Control	19**M** 19**F**	13.1 (2.1)	Residential Child Care	ET	14**M** 12**F**	12.7 (2.1)	Healthy General Population	Non-ET	Saliva/Elisa Kit	—	TSCC, IES-R	64.0
Nishi et al., 2015	Cohort	134**M** 51**F**	18-69	Inpatients Intensive care unit	RTE ^#^	—	—	—	—	Blood/ELISA Kit	—	IES-R	81.0
Reijnen et al., 2017	Cohort	907 **F/M**	28.38 (8.91)	Military after combat	ET PTSD	—	—	—	—	Blood/ ELISA Kit	—	ETIS-SR, DSRI	80.6
**2 - EXPERIMENTAL STUDIES - STRESS REACTIVITY**													**Mean: 87.83%**
Pierrehumbert et al., 2010	Pre/Post	13**M** 16**F** 20**M** 26**F**	33.96 (5.86) 25.08 (4.96)	Outpatients/ Health Healthy/ Cancer Survivors	ET^##^+ET^###^	14**M** 12**F**	29.63 (8.50)	Healthy General Population	Non-ET	Blood/RIA	Stress Test/ TSST-C	Interview	81.8
Munro et al., 2013	Pre/Post	15 **F**	18-35	Healthy University Students	ET/Non-ET / PTSD/Non-PTSD	—	—	—	—	Blood/RIA	Stress Test / Film Protocol	ELS PCL	89.0
Seltzer et al., 2014	Pre/Post	17**M** 21**F**	8-11.5	Maltreatment Child Protective Services	ET^###^	18**M** 36**F**	8-11.5	Heathy General Population	No ET	Urine/RIA	Stress Test/ TSST-C	PSC	92.7
**3 - CLINICAL TRIALS, RANDOMIZED, DOUBLE-BLIND, PLACEBO-CONTROLLED - EXOGENOUS ADMINISTRATION**													**Mean: 77.04%**
Meinlschmidt and Heim, 2007	Crossover	9 **M**	20-28	Healthy University Students	ET	10 **M**	20-28	Healthy University Students	Non-ET	OXT vs. PLA intranasal (24UI)	[Cortisol]/saliva [OXT] Blood	SSRQ	61.2
Grimm et al., 2014	Crossover	14**M**	21-37	Healthy General Population	ET	17**M**	21-37	Healthy General Population	Non-ET	OXT vs. PLA Intranasal (40UI)	fMRI/Brain activation Stress test/MIST [Cortisol]/saliva	CTQ	81.2
Fan et al., 2014	Crossover	18 **M**	21-36	Heathy General Population	ET	—	—	—	—	OXT vs. PLA intranasal (24UI)	fMRI/Brain connectivity Stress test*/* MIST Anxiety/ STAI	CTQ	77.4
Eidelman-Rothman et al., 2015	Crossover	28 **M**	<35	War Veterans Israel Defense Force	PTSD	16**M**	<35	War Veterans Israel Defense Force	Non-PTSD	OXT vs. PLA intranasal (24 UI)	MEG/Brain activation [OXT]/blood/saliva	PDS	71.8
Koch et al., 2016a	Crossover	21 **M** 16 **F**	18-65	Outpatients Police Personnel	PTSD	20**M** 20**F**	18-65	Heathy Police officers	RTE	OXT vs. PLA Intranasal (40 UI)	fMRI/Brain connectivity Facial Task	CAPS, SCID	74.1
Koch et al., 2016b	Crossover	21**M** 16 **F**	18- 65	Outpatients Police Personnel	PTSD	20**M** 20**F**	18-65	Healthy Police officers	RTE	OXT vs. PLA intranasal (40 UI)	fMRI/Brain activation Resting State	CAPS, SCID ETIS-SR	75.0
Palgi et al., 2016	Crossover	23**M** 9 **F**	22-60	Outpatients Healthy Institute	PTSD	19**M** 11F	21-59	Healthy General Population	Non-PTSD	OXT vs. PLA intranasal (24 UI)	Compassion task/listen four Recorded Stories	CAPS, SCID	77.4
Frijling et al., 2016a	Parallel Group	9 **M** 10 **F**	18-65	Outpatients Emergency Department	RTE	9**M** 9**F**	18-65	Outpatients Emergency Department	RTE	OXT vs. PLA intranasal (40 UI)	fMRI/Brain connectivity Accident images vs. neutral Images Task	CAPS, SCID-IV, PDI, TSQ, ETIS-SR	75.0
Frijling et al., 2016b	Parallel Group	9**M** 14**F**	18-65	Outpatients Emergency Department	RTE	8**M** 10**F**	18-65	Outpatients/ Emergency Department	RTE	OXT vsPLA intranasal (40 UI)	fMRI/Brain activation Facial Task/EFMT	CAPS, PDI, TSQ	74.1
Nawijn et al., 2016	Crossover	21 **M** 14 **F**	18-65	Outpatients Police Personnel	PTSD	19**M** 18**F**	18-65	Police officers	RTE	OXT x PLA Intranasal (40 UI)	fMRI/Brain connectivity PTSD Task Monetary/MID	CAPS, SCID	75.0
Van Zuiden et al., 2017	Parallel Group	53**F/M**	18-65	Trauma centers	RTE	54 **F**/**M**	18-65	Trauma centers	RTE	OXT x PLA intranasal (40 UI)/ 8 days	Depression/ HADS	CAPS, MNI, IES-R, ETIS-R	81.25
Sack et al., 2017	Crossover	35 **M**	39.8 (11.2)	Outpatients Psychosomatic Clinic	PTSD	10**F**	36.08 (8.56)	Healthy	Non-PTSD	OXT x PLA intranasal (24 UI)/ 2 weeks	Trauma Script Challenge/ RSDS/ HR	DSM-IV, SCID	100
Nawijn et al., 2017	Crossover	21**M** 19**F**	18-65	Outpatients Police Personnel	PTSD	20**M** 20**F**	18-65	Police officers	RTE	OXT x PLA intranasal (40 UI)	fMRI/Brain connectivity Social incentive delay task	CAPS, SCID	78.1
**4-OBSERVATIONAL STUDIES – ASSOCIATION WITH POLYMORPHISMS OXYTOCIN RECEPTOR GENE**													**Mean: 76.27%**
Bradley et al., 2011	Cross-sectional	1347**F/M**	36.36 (13.6)	Outpatients Primary Care	ET/Non-ET	—	—	—	—	OXTR 53576	Attachment/APQ Emotional Deregulation/EDS	CTQ, TEI	73.0
Cicchetti and Rogosch, 2012	Case-Control	313**F/M**	6-12	Healthy Sumer Camp Program	ET	282 **F/M**	6-12	Healthy Sumer Camp Program	Non-ET	OXTR 53576	Resilience/PEI	Interview	65.0
Lucas-Thompson and Holman, 2013	Cohort	704**F/M**	18-101	9/11 Attack	PTSD/ RTE Non-PTSD	—	—	—	—	OXTR 53576	—	PCL	84.0
McQuaid et al., 2013	Cross-sectional	213**F** 75**M**	19 (3.1)	Healthy University Students	ET/Non-ET	—	—	—	—	OXTR 53576	Depression/ BDI	CMQ	87.0
Hostinar et al., 2014	Case-Control	263 **F/M**	13-15	Maltreatment Child Protective Services	ET	162 **F/M**	6-12	Healthy General Population	Non-ET	OXTR 53576	Int/Ext/ YSR Social Support/NRI	Interview	81.0
Myers et al., 2014	Cross-sectional	306**F** 347**M**	37	Healthy General Population	ET/Non-ET	—	—	—	—	OXTR 139832701 11131147	Depression/ DASS	ELS	77.0
Dunn et al., 2014	Cohort	205**F/M**	18-34	Recent Trauma Hurricane Katrina	PTSD/ RTE Non-PTSD	—	—	—	—	OXTR 53576/ 2254298	—	PTG, IES-R	71.0
Dannlowski et al., 2016	Cross-sectional	309**F/M**	18-59	Healthy	ET/Non-ET	—	—	—	—	OXTR 53576	fMRI/Brain activation Facial Task/EFMT	CTQ	67.9
Tollenaar et al., 2017	Cohort	2567**F/M**	18-65	Healthy	ET/Non-ET	—	—	—	—	OXTR 2254298/ 53576/ 2268498	Depression and Anxiety/ DSM-IV/ CIDI	CTQ + Interview	75
Sippel et al., 2017	Cohort	153**M**	51.50(15.51)	War Veterans	PTSD	2010**M**	63.80(14.06)	Health	RTE /No PTSD	OXTR 53576	Attachment Style Questionnaire	DSM-IV; PLC	81.8

* Technique Used For Measuring and/or Test Kit And Dose And Via Administration;

** Quality Assessment According To STROBE, CONSORT, and TREND;

# , Current trauma by motor or vehicle accident;

## , ET by cancer survival in childhood;

### *;:ET by physical abuse; APQ, Attachment Prototype Questionnaire; BDI, Beck Depression Inventory; CAN, Canada; CAPS, Clinician Administered; CIDI, Composite Interview Diagnostic Instrument; PTSD Scale; CES, Center For Epidemiological Studies Depression Scale; CMQ, Childhood Maltreatment Questionnaire; Cross, Cross Sectional; CSF, Cerebrospinal Fluid; CTQ, Childhood Trauma Questionnaire; DSRI, Dutch Self-rating Inventory; DASS, Depression Anxiety and Stress Scale; DCP, Dependent Children Program; DHS, Department of Human Services; DSRSC, Depression Self Rating Scale For Children; DSM-IV, Diagnostic and Statistical Manual of Mental Disorders; EDS, Emotional Deregulation Scale; EFMT, Emotional Face Matching Task; ELS, Early Life Stress Questionnaire; ELS-A, Early Life Stressful Adolescence; ELS-C, Early Life Stressful Experiences In Childhood; EDS, Emotional Dysregulation Scale; ENG, England; EPS, Parental Early Separation; ET, Early Trauma; ETIS, Early Trauma Inventory; ETISR-SF, Early Trauma Inventory Self Report -Short Form; ETI, Early Trauma Inventory; fMRI, Functional Magnetic Resonance Imaging; F, Female; GER, Germany; IES, Impact of Event Scale Revised; IN/ EXT, Internalizing/ Externalizing Symptoms; HR, Heart Hate; ISR, Israel; JAN, Japan; KIVS, The Karolinska Interpersonal Violence Scale; MEG, Magnetoencephalography; MID, Monetary Incentive Delay; MIST, Montreal Image Task; NETH, Netherland; M, Male; MINI, Mini-International Neuropsychiatric Interview; NRI, Network Relationships Inventory; OXT, Oxytocin; PCL, Checklist Civilian Version for DSM-IV; PDI, Peritraumatic Distress Inventory; PDS, Post Traumatic Stress Diagnostic; PEI, Pupil Evaluation Inventory; PG, Parallel Group; PLA, Placebo; PSC, Parental Child Conflict Scale; PTG, Post Traumatic Growth; PTSD, Post Traumatic Stress Disorder; Rsles, Recent Stressful Life Events; RIA, Radioimmunoassay; RSLE, Recent Stressful Life Events; RSDS, Dissociative Symptoms Scale; RTE, Recent traumatic experiences; SCID, Clinical Interview And Structured (DMS-IV); SDS, Social Disability Scale; SSRQ, Standardized Self Report Questionnaire; STAI, State-Trait Anxiety Inventory; SWI, Switzerland; TEI, Traumatic Events Inventory; TSCC, Trauma Symptoms Check List For Children; TSQ, Trauma Screening Questionnaire; TSST-C, Trier Social Stress Test; UI, International Units; USA, United States of America; YSR, Youth SelfReport; [OXT], Oxytocin Concentration Endogenous; 9/11 Attack = terrorist attack USA*.

Importantly, regarding the methodological quality evaluation, all of the studies included in this review had at least 64% of their essential items included in the STROBE (observational studies), TREND (experimental studies) or CONSORT [experimental or randomized controlled trials (RCTs)].

Depending on their designs or objectives, the studies were divided into four distinct groups: (a) observational studies evaluating endogenous OXT levels; (b) experimental studies related to the reactivity of the oxytocinergic system; (c) RCTs of OXT administration and the experience of either ET, RTE, or PTSD; and (d) observational studies investigating the effect of polymorphisms of the OXT receptor gene.

The major results of each study group are presented below.

(a) Observational studies evaluating endogenous OXT levels.

The association between endogenous OXT levels and ET situations was evaluated by six studies as a major outcome. Four of these studies found significant correlations between endogenous OXT levels and ET, with values ranging from −0.54 to −0.23 (Heim et al., 2009; Opacka-Juffry and Mohiyeddini, 2012; Chatzittofis et al., 2014; Mohiyeddini et al., 2014). On the contrary, Mizushima et al. (2015) did not find an association between OXT levels and experiencing ET (*p* >0.05; *d* = 0.19; insignificant effect size). Importantly, however, OXT secretions were markedly increased in adolescents who suffered abuse and lived in a stable environment (e.g., a social welfare institution) at the time of the experiment from awakening to bedtime compared with those who had a history of abuse and lived in unstable environments. Mizuki and Fujiwara (2015) showed that only less severe forms of ET were associated with increased OXT levels.

Heim et al. (2009) also performed complementary analyses to evaluate the effect of the recurrence of traumatic events. These authors found that experiencing three or more types of traumatic events during childhood was related with reduced endogenous OXT levels, with a moderate effect size (n_*p*_^2^ = 0.45). Opacka-Juffry and Mohiyeddini (2012) also conducted complementary analyses and found associations between decreased endogenous OXT levels and increased emotional suppression (r = −0.30, *p* < 0.01) during adulthood.

Studies that evaluated participants with RTEs failed to find any significant correlations. For example, Opacka-Juffry and Mohiyeddini (2012) found that the correlation between OXT and RTE was 0.01 (*p* > 0.05). Chatzittofis et al. (2014) also failed to find an association between endogenous OXT levels and RTEs (*r* = −0.30, *p* = 0.18); however, these authors found that endogenous OXT levels were lower in individuals with RTEs who also experienced ET (i.e., were re-victimized) than in those who only experienced trauma during childhood (*p* = 0.04).

Nishi et al. (2015) also failed to find an association between PTSD symptoms and endogenous OXT levels (*r* = −0.08 to −0.00, *p* > 0.57). However, these authors showed interesting differences between the genders: In women, endogenous OXT levels were positively correlated with cooperativeness (*r* = 0.41, *p* = 0.01), whereas in men these levels were negatively correlated with C-reactive protein (*r* = −0.22, *p* < 0.01), which indicates that OXT plays a role in the coping strategies for PTSD symptoms among women.

The findings of Frijling et al. (2015) also indicated gender differences. Regarding endogenous OXT levels in highly traumatized police officers, only men with PTSD showed lower levels of OXT than those without PTSD (*p* < 0.05, *d* = 0.60; moderate effect size). No differences were observed in women (*p* > 0.05; *d* = 0.10; insignificant effect size).

Finally, a recent study by Reijnen et al. (2017) found that pre-deployment OXT levels in soldiers sent to Afghanistan did not predict PTSD development. However, the experience of an ET predicted the development of PTSD, even though no associations were found between OXT levels and the presence/absence of ET in these individuals.

(b) Experimental studies relating to the reactivity of the oxytocinergic system.

Three studies evaluated OXT levels (reactivity patterns) in acute stressful situations, although these results should be interpreted with caution because of their small sample sizes (Table 2).

**Table 2:** Major results of endogenous OXT during experimental studies (reactivity to stress) of participants who experienced trauma (*n* = 3).

**Study**	**Type of stress test**	**Results**			
		**[OXT] situation**	**Reactivity to stress**	***r***	***p***
Pierrehumbert et al., 2010	TSST-C	Pre-Stress [OXT]	ET sexual abuse = ET cancer childhood = control	—	0.23
		Post-Stress (+20 min) [OXT]	ET sexual abuse < control	—	0.06
Seltzer et al., 2014	TSST-C	Post- Stress (+ 30 min) [OXT]	♀ ET physical abuse > ♀ control	—	0.02^*^
			♂ ET physical abuse = ♂ control		0.07
Munro et al., 2013	Film Protocol (Abandonment and Bond scenes)	Bonding [OXT]	Basal → Bonding = no alterations Dissociation symptoms x higher levels [OXT]	0.55	0.39 0.018^*^
			Somatization symptoms x higher levels [OXT]	0.59	0.010^*^
		Abandonment [OXT]	Basal → Abandonment = ↓	—	0.01^*^
			PTSD x lower levels [OXT]	0.35	0.010

[OXT], oxytocin; ET, Early trauma; PTSD, Post-traumatic stress disorder; ♂, Men; ♀, Women; TSST-C, Trier Social Stress Test; < Minor; > Major

* *Difference statistically significant*.

Girls who experienced physical abuse during childhood showed increased reactivity to stress. In other words, they presented with higher levels of endogenous OXT after undergoing an acute stress test (*p* = 0.02), whereas OXT levels in boys with and without a history of physical abuse did not change after stress induction (Seltzer et al., 2014). On the other hand, adults with a history of sexual abuse (regardless of gender) showed decreasing OXT levels after acute stress (Pierrehumbert et al., 2010).

Munro et al. (2013) also found a decrease in OXT levels after exposure to abandonment scenes (*p* = 0.01) but not after exposure to bonding scenes (*p* = 0.39). However, associations were observed between increased OXT levels and increased dissociative, somatic, and attachment symptoms during bonding scenes as well as between decreased OXT levels and PTSD symptoms during abandonment scenes.

(c) RCTs of OXT administration and trauma.

The results of these trials were grouped by the type of trauma experienced and are shown in Table 3.

**Table 3:** Major results of RCTs that administered OXT to participants who experienced early or current trauma *(N* = 13).

**Study**	**Outcomes**	**Main results**		
		**Treatment**	**Comparison groups**	**Brain activation/ Connectivity/[cortisol]/ Symptomatology results**
**(1) EARLY TRAUMA**				
Meinlschmidt and Heim, 2007	[Cortisol]	OXT vs. Placebo	ET / Non-ET	Attenuated the 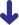 [cortisol] after OTX in subjects with ET
Grimm et al., 2014	Brain activation fMRI/ 3T Voxel wise MIST (Stress task) [Cortisol]	Placebo OXT OXT vs. placebo	ET vs. Non-ET Non-ET ET ET vs. Non-ET Non-ET ET ET Non-ET	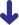 Activation left hippocampus and dorsomedial thalamus 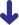 [cortisol] after stress task 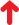 [cortisol] after stress task 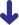 Activation at right insula, anterior ACC, PCC, left parahippocampal gyrus No effect on the [cortisol] after stress task 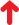 [cortisol] after stress task 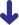 **Hormonal limbic reactivity** (pgACC, left amygdala, left parahippocampal gyrus, left insula, bilateral putamen and bilateral caudate), during the stress test 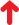 **Hormonal limbic reactivity** (pgACC, left amygdala, left parahippocampal gyrus, left insula, bilateral putamen and bilateral caudate), during the stress test
Fan et al., 2014	Brain connectivity fMRI/ 3T Voxel/ wise Seed/based approach Resting state MIST (Stress task)	Placebo OXT OXT x ET Placebo OXT	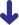 (severity)ET^*^ 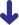 (severity)ET^*^ 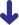 (severity)ET^*^ ET ^*^ & pgACC-amygdala rs/FC 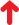 ET^*^ & an increased pgACC-amygdala rs/FC 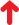 ET^*^ & an increased pgACC-amygdala rs/FC 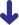 ET^*^ 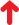 ET^*^ 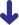 ET	**Resting state** 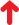 pgACC-amygdala rs/FC It was not significant effect of OXT It was no significant effect of OXT & ET interaction **Psychosocial Stress** Correlated with state anxiety, that correlate with [cortisol] Negatively predicted anxiety Predicted stronger pgACC deactivation during stress Predicted weaker pgACC deactivation Attenuated rest-task interaction between pgACC-amygdala rs/FC and pgACC deactivation
**(2) CURRENT TRAUMA**				
Frijling et al., 2016a	Brain connectivity fMRI/ 3T Voxel/bold Accident images vs. Neutral images Task	Placebo OXT OXT vs. Placebo	RTE RTE	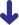 Connectivity left amygdala and PFC for trauma images vs. neutral images 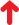 Connectivity left amygdala and PFC for trauma images vs. neutral images 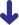 Amygdala connectivity to the left insula for trauma images vs. neutral images 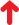 Amygdala connectivity vmPFC for trauma images vs. neutral images 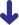 Flashbacks of memory during accident images
Frijling et al., 2016b	Brain activation fMRI/ 3T Voxel/bold Facial task	OXT vs. Placebo	RTE	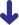 BL amygdala activation for fearful faces ♂ 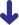 Left amygdala to neutral faces
Van Zuiden et al., 2017	PTSD symptoms	OXT vs. placebo	**High PTSD symptoms** **Low PTSD symptoms**	OXT < Placebo OXT = Placebo
**(3) PTSD**				
Eidelman-Rothman et al., 2015	MEG Frequency/Hz Alpha Resting State activation	Placebo OXT	PTSD vs. Non-PTSD PTSD vs. Non-PTSD PTSD and Non-PTSD	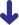 α resting-state activity in left dPFC, SFG and MFG 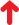 α resting-state activity in left SFG e MFG 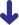 α resting-state activity in left SFG and MFG correlated with re-experience symptoms
Palgi et al., 2016	Compassion task	OXT vs. placebo	PTSD	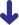 Compassion toward women protagonist No effect on compassion toward masculine protagonist
Koch et al., 2016a	Brain activation fMRI/ 3T Voxel/bold Facial task	Placebo OXT OXT vs. placebo	PTSD RTE PTSD RTE RTE PTSD	Valence-dependent amygdala reactivity was absent for the left amygdala 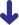 Reactivity of amygdala to fearful-angry faces compared with happy -neutral faces 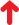 Reactivity of left amygdala all emotions 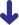 Increased reactivity of the left amygdala 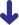 Activation of left amygdala for all emotions 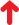 Activation of left amygdala
Koch et al., 2016b	Brain connectivity fMRI/ 3T Voxel/bold Resting State	Placebo OXT	♂PTSD vs. ♂ RTE ♀PTSD vs. ♀ RTE ♂PTSD vs. ♂ RTE ♀PTSD vs. ♀ RTE PTSD	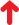 Connectivity right CeM amygdala to left vmPFC 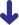 Connectivity right BLA to bilateral dACC 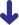 Connectivity right CeM to left vmPFC 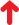 Connectivity right BLA to right dACC 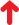 Anxiety and nervousness but not happiness and sadness
Nawijn et al., 2016	Brain activation fMRI/ 3T Voxel/bold Task/Monetary	Placebo OXT vs. placebo	PTSD and RTE Non-PTSD	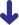 Reaction time on MID task for reward/loss vs. neutral trials 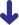 Brain activation at ventral striatum, amygdala, insula, CPF orbitofrontal 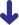 Brain activation at right striatum, dACC, and insula during reward and loss
Sack et al., 2017	Trauma Script Challenge	OXT	PTSD	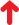 reduced avoidance symptoms No effect on re-experiencing and dissociative symptoms 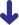 Heart rate
Nawijn et al., 2017	Brain activation fMRI/ 3T Social Task	Placebo OXT OXT vs. Placebo	PTSD vs. RTE Non-PTSD PTSD vs. RTE Non-PTSD PTSD	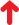 Activation insula anterior left during social incentive 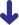 Activation bilateral putamen, right dACC and right insula during social incentive 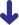 Activation right Putamen during social incentive 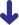 Activation left striatum; right striatum and insula, and right dorsal ACC

* *Early trauma of emotional abuse; α, alpha; ACC, Anterior cingulate cortex; BLA, Amygdala basolateral; BL, Basolateral; CeM, central medial amygdala; CAPS, Clinician-administered PTSD Scale; DMPFC, Dorsomedial pre-frontal cortex; dACC, Dorsal anterior cingulate cortex; dPFC, Dorsolateral pre-frontal cortex; MFG, Middle frontal gyrus; MEG, Magnetoencephalography; MID, Monetary incentive delay task; MIST, Montreal Image Task; PTSD, Post traumatic stress disorder; OXT, Oxytocin; rs/FC, resting state/ functional connectivity; VmPFC, Ventral medial pre frontal cortex; VlPFC, Ventral lateral pre frontal cortex; SFG, Superior frontal gyrus; MFG, Middle frontal gyrus; ET, Early trauma; [Cortisol], concentration of cortisol; fMRI, Functional magnetic resonance imaging; pgACC, Pregenual cortex cingulate anterior; RTE, Recent traumatic experiences; SPG, superior frontal gyrus; sgACC, Subgenual anterior cingulate cortex*.

Three studies evaluated participants with just ET. In the first, Meinlschmidt and Heim (2007) measured endogenous cortisol and demonstrated that intranasal OXT attenuated the cortisol decrease in participants with ET compared with controls, suggesting the presence of amortization effects related to HPA-axis activities. In the other two studies, participants with ET were assessed in the context of a psychosocial stress situation, and the results indicated that negative outcomes were associated with acute OXT administration.

In one of these studies, Grimm et al. (2014) found that participants with ET, regardless of severity, presented with greater hormonal and limbic reactivity after the use of OXT. However, Fan et al. (2014) found that the activation of the connectivity between the amygdala and the pregenual anterior cingulate cortex (pgACC) during stress was only attenuated by OXT in individuals with less severe ET. The use of OXT did not favor this anxiolytic effect in patients with moderate-to-severe ET.

Two studies by Frijling et al. (2016a,b) reported the adverse effects of exogenous OXT administration in individuals with RTEs. In one such study (Frijling et al., 2016a), OXT administration during threatening situations reduced the functional connectivity between the left amygdala and the ventrolateral prefrontal cortex (vlPFC) as well as between the amygdala and the ventromedial prefrontal cortex (vmPFC) circuits responsible for cognitive-emotional regulation and fear extinction. These findings were also accompanied by an increased connectivity between the amygdala and the insula as well as increased episodes of traumatic flashbacks. In the other study (Frijling et al., 2016b), increased amygdala reactivity was elicited by fearful faces, indicating that OXT favors an increase in the processing of fear salience and, consequently, anxiogenic effects.

A single study administered OXT for eight subsequent days and showed that it did not attenuate PTSD symptoms in the short term (45 days). However, only participants with high symptom severity reported improvement after 6 months, which suggests that OXT may has a protective effect in the long term which is mediated by symptom severity (Van Zuiden et al., 2017). In this study, as well as at Fan et al. (2014), it is evident the influence of the severity of experience/symptoms of trauma.

Contrary to the effects observed in subjects with RTE, in those with a PTSD installed, the related effects to acute OXT administration were favorable. Eidelman-Rothman et al. (2015) examined veterans with PTSD and showed that OXT normalized the resting-state brain functioning of these individuals, which was similar to those of controls (i.e., veterans not exposed to trauma). Prior to exogenous OXT administration, an increase in resting-state alpha activity was observed in the left dorsolateral prefrontal cortex (dPFC), especially in the superior frontal gyrus (SFG) and middle frontal gyrus (MFG). These regions are associated with memory and cognitive control, which are important for emotional control.

Koch et al. (2016a,b) and Nawijn et al. (2016, 2017) examined the same sample of participants (i.e., trauma-exposed police officers with or without PTSD), in different paradigms outcomes and showed positive effects during acute OXT administration. In a resting state paradigm the acute OXT effects were different between genders. In men with PTSD, OXT decreased subjective anxiety and nervousness as well as restored the connectivity between right amygdala (CeM) and the left vmPFC. In women, OXT restored the connectivity between the right basolateral amygdala (BLA) and the anterior cingulate dorsal cortex (dACC), which decreased the anxiety and fear expression originating from the amygdala (Koch et al., 2016b). In an emotional face-matching task, the acute effects of OXT in subjects with PTSD were the same, regardless of gender: OXT reduced amygdala reactivity to all emotional expressions. In those without RTE, however, reactivity was increased, which indicates the presence of the anxiolytic effects of OXT only in trauma-exposed individuals who develop PTSD (i.e., an interdependence of inter-individual factors; Koch et al., 2016a). During a monetary task, OXT increases neural responses during anticipation of reward or loss in key regions of the brain's reward circuit (i.e., the striatum, dACC, and insula) and decreases motivational anhedonia. These effects were positively associated with those of OXT in the ventral striatum (Nawijn et al., 2016). Similarly, during a social incentive delay task, the administration of OXT normalized the aberrant insula response and increased the putamen response, indicating increased neural sensitivity to social reward (Nawijn et al., 2017). Finally, it was observed as positive effects related to OXT, an increase in compassion toward women with PTSD (Palgi et al., 2016) and a decrease in the avoidance symptoms during trauma script exposure (Sack et al., 2012).

(d) Observational studies investigating the effect of the polymorphisms of the OXT receptor gene.

The major polymorphism studied was OXTR rs53576, which was evaluated in 88.8% of the included studies. Table 4 shows the main results found.

**Table 4:** Main results of the association studies of early or current trauma with regard to OXT receptor gene polymorphisms (*N* = 10).

**Study**	**Phenotype**	**Results genetic association with ET, RTE and PTSD**			**Statistics**	
					***P***	**Effect size**
**POLYMORPHISM OXTR rs53576**						
Bradley et al., 2011	Emotional deregulation Problematic attachment	3 ET or more Non-ET ET Non-ET		GG > AA/AG GG = AA/AG GG > AA/AG GG = AA/AG	<0.001 0.49 0.02 0.05	*d* = −0.052
Cicchetti and Rogosch, 2012	Resilience	ET+AA/AG ET+GG	<<	Non-ET +AA/AG Non-ET +GG	< 0.01 0.02	n*_*p*_*^2^ = 0.083 n*_*p*_*^2^ = 0.01
Lucas-Thompson and Holman, 2013	PTSD symptoms	RTE^*^+GG RTE^*^+GA/AA RTE^**^+GG RTE^**^+GA/AA	> = > >	Non-RTE^*^+GG Non-RTE^*^+GA/AA Non-RTE^**^+GG Non-RTE^**^+GA/AA	< 0.01 0.12 < 0.01 < 0.01	
McQuaid et al., 2013	Depression symptoms	Low ET+GG/GA High ET+GG/GA	= >	Low ET+AA High ET+AA	0.07 < 0.01	
Hostinar et al., 2014	Perception of social support Internalizing behavioral problems Externalizing behavioral problems	ET+GG Non-ET+GG ET+GG ET+GG	< = > =	ET+GA/AA Non-ET+GA/AA ET +AA/AG ET +AA/AG	0.02 0.35 0.01 0.11	n*_*p*_*^2^ = 0.02 n*_*p*_*^2^ = 0.02
Dunn et al., 2014	PTSD symptoms PTSD development		GG = AG = AA GG = AG = AA		0.90 0.70	
Dannlowski et al., 2016	Ventral striatum gray matter volume Enhanced activation of amygdala to positive and negative emotional	ET+GG Non-ET+GG/GA ET/Non-ET+GG	< > >	AA AA ET/Non-ET+ AA	< 0.01 >0.05 < 0.05	
Tollenaar et al., 2017	Depression symptoms Anxiety symptoms	ET/Non-ET	GG = AG = AA GG = AG = AA		>0.90 >0.86	
Sippel et al., 2017	Insecure attachment style	PTSD	A allele + insecure attachment = risk factor for PTSD		0.02	
**POLYMORPHISM OXTR rs2254298**						
Dunn et al., 2014	PTSD symptoms	RTE+GG = RTE+AG = RTE+AA			0.45	
	PTSD development	RTE+GG = RTE+AG = RTE+AA			0.85	
**POLYMORPHISM OXTR rs2254298** + **rs2268498**						
Tollenaar et al., 2017	Depression Symptoms Anxiety Symptoms	ET/Non-ET	GG = AG = AA GG = AG = AA	>0.08 >0.21	– –	
**POLYMORPHISM OXTR rs139832701** + **rs11131147**						
Myers et al., 2014	Depression symptoms Stress symptoms	ET >Non-ET ET >Non-ET			0.004 0.0016	

* Recent traumatic experiences by economic stress;

** *Recent traumatic experiences by negative environment; A, A allele; d, Cohen's d; ET, early trauma; G, G allele; ${\text{n}}_{p}^{2}$, partial eta squared; PTSD, post-traumatic stress disorder; p, level of significance; Non-ET, absence of early trauma; RTE, Recent traumatic experiences*.

Regarding the results linked to the OXTR rs53576 gene polymorphism, participants who exhibited the GG genotype and experienced ET, PTSD, or both reported a series of impairments including emotional dysregulation and problematic attachment (Bradley et al., 2011), PTSD symptoms (Lucas-Thompson and Holman, 2013), symptoms of depression (McQuaid et al., 2013), lower levels of perceived social support, and internalized behavioral problems (Hostinar et al., 2014). Using structural magnetic resonance, Dannlowski et al. (2016) showed that the presence of the G allele (GG/GA) was associated with increased amygdala responsiveness to all emotional facial expressions (negative and positive), constituting a higher vulnerability to alterations in the limbic brain structure in individuals with ET. Furthermore, a negative correlation was found between ventral striatum gray matter volume and participants with ET and the GG genotype.

Cicchetti and Rogosch (2012) showed that the presence of ET and the AA/AG genotype was associated with increased resiliency; therefore, the presence of the AA/AG genotype appears to have a protective function. On the other hand, Tollenaar et al. (2017) indicated that the OXTR53576 gene polymorphism does not interact with ET and predict risk factor and/or vulnerability toward the development of depression or anxiety. In addition, Sippel et al. (2017) found that the presence of a minor allele associated with an insecure attachment style was associated with a higher prevalence of PTSD among war veterans.

Other polymorphisms studied included OXTR rs2254298 and rs2268498, but neither Dunn et al. (2014) nor Tollenaar et al. (2017) found changes or association among the presence of ET or PTSD, a single nucleotide polymorphism, and the development of symptoms. In addition, Dunn et al. (2014) investigated the role of rs53576 and did not find a difference between the presence of the AA allele and the AG or GG alleles as risk factors for the development of PTSD or its symptoms.

Finally, one study evaluated the polymorphisms OXTR rs139832701 and rs11131147. This study showed that participants who experienced ET and had these polymorphisms exhibited higher levels of depression and more symptoms of stress (Myers et al., 2014).

## Discussion

The present review revealed associations between traumatic experiences in humans and the neuropeptide OXT that involve polymorphisms on OXT receptor genes. These associations are multiple and complex and are mediated by contextual and inter-individual factors. A stronger association with OXT was found with regard to the experience of early or chronic trauma as well as with recurrent, severe, or intense traumatic events. Regarding RTEs, the association was strong among individuals who developed psychopathological conditions (e.g., PTSD).

The findings of the group of studies that evaluated endogenous OXT levels showed a moderate association between reduced OXT levels and the experience of trauma. This finding supports the hypothesis that early adversity persistently, even up to adulthood, alters the functioning of the suprachiasmatic nucleus, which is responsible for the production and release of OXT (Ozbay et al., 2008; Gonzalez et al., 2009; Nicolson et al., 2010; Goldman-Mellor et al., 2012). This change might favor vulnerability to stress during adulthood and impair the social functioning associated with OXT. Moreover, it might reduce coping and resiliency responses (Opacka-Juffry and Mohiyeddini, 2012; Frijling et al., 2015). Interestingly, Munro et al. (2013) showed that an increase in endogenous OXT was associated with symptoms of social detachment, which might not always result in favorable outcomes (Seng, 2010).

However, an increase in endogenous OXT occurred in participants with ET and specific conditions such as less severe forms of ET and social environmental change (from a threatening environment to a protective environment with the establishment of stable social relationships). This finding shows the role of OXT in the development and maintenance of resiliency (Elzinga et al., 2008; Carpenter et al., 2009; Heim et al., 2009). Authors such as Mizuki and Fujiwara (2015) and Mizushima et al. (2015) suggested that an increase in OXT occurs as a response to social stress, thereby promoting the regulation of the oxytocinergic system and an increase in pro-social behaviors under challenging and less unfavorable situations. These findings also reinforce Veenema (2012) views concerning the high plasticity of the oxytocinergic system and its dependence on social cues.

Studies have indicated the mediating role that other variables play in traumatic situations during adulthood because OXT is reduced only in subjects with ET and/or PTSD, demonstrating decreased resiliency responses. It has also been suggested that stress affects the oxytocinergic system in a sex-dependent manner (see especially Nishi et al., 2015), which has been extensively documented in animals (Ebner et al., 2000; Cameron et al., 2008).

The studies related to stress reactivity have also shown gender dependence because only women with a history of physical abuse show increased OXT secretion under acute stress (Seltzer et al., 2014). When gender was not experimentally controlled, a decrease in OXT secretion was observed (Munro et al., 2013). Previous studies have shown that men and women differ with regard to the release of endogenous OXT following behavioral paradigms. They also differ behaviorally after exogenous OXT administration (Finkelhor et al., 1990; Heim et al., 2000; Seng et al., 2014; Feng et al., 2015; Koch et al., 2016a,b).

One possible explanation of the above gender effects might involve the biochemical differences in the number of available OXT receptors between men and women as well as the binding affinity of these receptors in specific neural networks (Uhl-Bronner et al., 2005; Hoge et al., 2014). Furthermore, the different phases of the menstrual cycle can also influence OXT levels as well as the HPA axis, conferring more or less impairment (Altemus et al., 2001). This effect is seen because of the role of estrogen, which regulates the production of OXT receptors and the possible release of this hormone (Williams et al., 1985; Wigger and Neumann, 1999). In contrast, evidence also shows that androgens inhibit OXT release under stress (Young et al., 1997). According to Taylor et al. (2006), OXT might also favor the “tend and befriend” response in women and the “fight or flight” response in men.

The results of the studies that focused on exogenous OXT administration varied by the type of stressor. Regarding ET, the effect of this stressor on the functioning of the brain circuits was demonstrated. ET favored functional changes in the brain circuits, especially those associated with limbic regions (e.g., the hypothalamus, pgACC, amygdala, and parahippocampal gyrus), which are HPA axis-modulating regions. In this sense, OXT modulates the neural networks to favor an improved response to stress (Meinlschmidt and Heim, 2007).

Other studies that examined severe ET, however, seemed to show a null or differential response to exogenous OXT administration that assumes anxiogenic characteristics. This effect might occur because of previous changes in the oxytocinergic circuit as a consequence of the traumatic experience or because of unregulated interactions between the oxytocinergic system and the other neurotransmitter systems (Fan et al., 2014; Grimm et al., 2014).

The effects of exogenous OXT in individuals with RTE at risk for PTSD were unfavorable because they tended to promote anxiogenic effects and did not reduce the risk of developing different pathologies, including PTSD. The results generally showed that OXT favors fear-related responses; this fact is also an accepted finding in studies conducted with healthy participants (Domes et al., 2010).

The above inconsistency can be understood in light of Heinrichs et al. (2004). These authors argue that OXT can produce an extinction effect on aversive memories among healthy participants, depending on the test type used and the relevance of the stimuli to the evaluated patient. According to their findings and the previous literature (Kirsch et al., 2005; Domes et al., 2007; Gamer et al., 2010), the effects of OXT on amygdala reactivity might differ not only because of inter-individual factors such as gender and level of psychopathology but also because of the context (i.e., the emotional content/valence of the stimulus).

The increased number of flashbacks suggests that the administration of OXT in individuals who experienced recent trauma impedes the functioning of the emotional regulation network in response to exposure to situations reminiscent of the trauma. This finding is contrary to the previous literature (Koch et al., 2016b; Sack et al., 2017) showing the potential role that OXT plays in the extinction of traumatic memories. In previous studies, postpartum women (i.e., those with increased OXT production) experience temporary deficits in memory that contribute to the extinction of the aversive memories associated with childbirth (Brindle et al., 1991; Brett and Baxendale, 2001).

On the other hand, the continuous use of OXT for 8 days had positive effects only for individuals with severe symptoms/traumas, again suggesting an interdependence of inter-individual variables. Thus, the studies of the use of OXT to prevent PTSD suggest the need for caution because of the possible negative/anxiogenic effects on the one hand and the presence of benefits resulting from OXT administration that occur selectively only in highly symptomatic individuals on the other.

Finally, for individuals with PTSD, the effects of acute OXT administration were favorable. Considering that PTSD is associated with hyperactivity in the amygdala and other limbic brain structures when presented with negative emotional stimuli (Brunetti et al., 2010; Frijling, 2017), OXT favored the reestablishment of the functioning of different neural networks associated with fear control and extinction responses, thereby favoring better emotional control and cognitive performance through the reduction of hypervigilance, avoidance, anhedonia, and emotional salience to fear.

Importantly, these studies showed weak effects for the control groups that were composed mostly of participants with RTEs (i.e., those exposed to trauma but without PTSD). This result reinforces the findings of the previous group of studies that indicated the lack of benefits for this specific sample. These data also reinforce the positions of Koch et al. (2016a,b) and Bartz et al. (2010) who argued that OXT is beneficial only for individuals with impaired fear regulation and social functioning. However, Palgi et al. (2016) examined participants exposed to trauma, with or without PTSD, and found that they benefited from the administration of OXT in terms of their compassion response.

Regarding the studies related to the different polymorphisms, the present review suggested that variations and polymorphisms are intrinsically associated with the changes in the stress resilience mechanism and the experience of traumatic experiences (Cicchetti and Rogosch, 2012). One possible explanation is that individuals with polymorphic variations in the OXT receptor gene due to random changes in the position of the amino acids are more vulnerable to the development of disorders resulting from traumatic experiences throughout the lifespan (Feldman et al., 2016).

The genetic polymorphisms that showed more damage were those associated with the GG genotype at the OXT genetic receptors rs53576, rs139832701, and rs11131147. These genetic variations might alter receptor configuration and develop changes at density, alter the number of receptors available in the central nervous system (Champagne and Curley, 2009; Skuse and Gallagher, 2011), and might develop alterations on OXT binding affinity to the receptor, thereby altering the effects/functions of OXT in the body.

Interestingly, Dannlowski et al. (2016) found that the presence of the GG genotype allied with ET might increase the vulnerability to the greater activation of limbic areas during the visualization of positive/negative faces; however, these authors indicated that such a vulnerability would also depend on environmental experiences and the complex interaction between genes and environment. Therefore, stable environments should provide more positive signals and lead to beneficial development, whereas threatening environments should result in detrimental effects on emotional development and lead to vulnerability to psychiatric disorders.

Champagne and Curley (2009) also suggested that participants exposed to stressful environments undergo DNA methylation processes that modify the positions of nitrogenous bases, thereby resulting in the modification of certain gene expression. Therefore, the binding affinity of OXT to the receptor might be altered, thereby reducing the amount of bound OXT and leading to a reduction in the function of OXT in the organism. This effect would alter the individual's vulnerability to the effects of stress (Jack et al., 2012).

On the other hand, one study (Sippel et al., 2017) indicated that polymorphisms on the OXTR rs53576 receptor gene with the presence of a single A' allele might contribute to the formation of insecure attachments among individuals with PTSD. This finding is relevant because this attachment style is associated with a reduced response to PTSD treatment. Such dysfunction might translate into a neural mechanism that could predispose individuals to negative assessments of their social environment stimuli, which might compromise the stress-reducing effects of social support and exacerbate the effect of cognitive representations of relationships as threatening, mistrustful, or unstable.

Finally, the other genetic polymorphisms (OXTR rs2254298 and rs2268498) had no effect on PTSD symptoms or their development (Dunn et al., 2014) nor on the depression or anxiety symptoms of participants with ET (Tollenaar et al., 2017). One possible explanation is that the participants were recruited from a specific population of low-income non-Hispanics blacks who were exposed to Hurricane Katrina (Dunn et al. 2014) or healthy participants with ET (Tollenaar et al., 2017). Large samples are required for candidate gene studies.

## Concluding remarks

This systematic review regarding the relationship between OXT and emotional trauma in humans revealed that reductions in endogenous OXT levels are more strongly associated with the presence of severe and recurrent ET and PTSD.

The results also show that the acute effect of OXT in victims of ET tends to be anxiolytic, but only in those with less severe forms of ET. Among individuals with severe ET, OXT does not seem to have anxiolytic properties and may even increase anxiety. The same holds true for victims of RTE, since OXT has been reported to increase the re-experience of traumatic symptoms. In patients with PTSD, OXT reestablished the function of different neural networks associated with fear control and extinction.

Genetic studies showed that the presence of the GG genotype in the OXTR rs53576 gene polymorphism associated with the experience of ET or PTSD is related to more negative outcomes, whereas the presence of the AA genotype appears to have a protective role.

It should be noted that the relationship between OXT and traumatic situations is mediated by different contextual and inter-individual variables that might predict more or less favorable outcomes. Thus, a stable environment, less severe forms of trauma, and the female gender are associated with resiliency responses.

The studies included in the review have limitations that call for caution in the interpretation and generalization of their findings, including (a) small sample sizes recruited from extremely specific contexts; (b) poor control of confounding variables such as gender and ET severity; (c) highly variable age groups; (d) cross-sectional designs; (e) possible differences between chronic and acute administration of OXT in RCT; and (f) lack of evaluation of both central and peripheral levels of OXT to determine whether differences exist [most of the studies on this topic assessed endogenous levels in peripheral fluids (e.g., blood plasma, urine, saliva) and the methodological validity of these techniques is controversial (Wotjak et al., 1998; Seltzer et al., 2014; Valstad et al., 2017)].

For future studies, we suggest the adoption of longitudinal designs to establish causal relationships; the recruitment of large samples to control for gender, age, and sex hormones; the conduction of RCTs with chronic and/or prolonged administration of OXT; the investigation of other polymorphisms that might interfere with or contribute to increased susceptibility to the harmful effects of traumatic experiences; and the performance of epigenetic investigations on the association between ET, RTE, and PTSD with OXT.

## Author contributions

All authors listed have made a substantial, direct and intellectual contribution to the work, and approved it for publication.

### Conflict of interest statement

The authors declare that the research was conducted in the absence of any commercial or financial relationships that could be construed as a potential conflict of interest.
